# Vertical displacement of pleura: a new method for bronchospasm evaluation?

**DOI:** 10.1186/s13089-020-00184-5

**Published:** 2020-08-25

**Authors:** Sara Raquel Martins, Ramon Nogué

**Affiliations:** 1 Centro Hospitalar Universitário do Porto, Porto, Portugal; 2 Universitat de Lleida, Lleida, Spain

**Keywords:** Asthma/COPD, Bronchospasm, Diagnostic imaging, Emergency medicine, Lung ultrasonography, Point-of-care ultrasonography

## Abstract

**Background:**

Lung ultrasonography has been increasingly recognized has a valuable diagnostic tool. In adult patients with asthma/chronic obstructive pulmonary disease and wheezing, LUS usually presents as an A/nude profile (normal profile, with sliding and A-lines, and without any abnormal findings) or at most reveals a decrease/absence of lung sliding. Therefore, until now simple point-of-care ultrasonography appeared to be unable to assess the severity of airflow limitation.

**Case presentation:**

We report the case of a woman presenting to the emergency department with an asthma exacerbation. Bedside ultrasound showed the usual A/normal profile, but also an associated vertical pleural displacement, probably secondary to hyperinflation and accessory muscle recruitment. We evaluated the described movement with M-mode and established a comparison index between end-inspiration and end-expiration, using the skin as reference. This index showed improvement and complete normalization during treatment.

**Conclusions:**

Pleural vertical displacement appears to be a sonographic alteration associated to bronchospasm and accessory muscle recruitment. It is easily identifiable and measurable on LUS, thus possibly representing a new method to evaluate bronchospasm and monitoring treatment response. Further research is needed to confirm or refute this finding.

## Introduction

Over the past few years lung ultrasonography (LUS) has been increasingly recognized has a valuable diagnostic tool. Ultrasound innocuousness, combined with its fast learning curve, portability and low cost have resulted in its ubiquitous use in almost any setting [1–6]. International evidence-based recommendations for point-of-care lung ultrasound, published in 2012, set foundations to a more regulated use [7]. Since then, several studies reported its applications in a large range of medical conditions.

LUS relies on the interpretation of four fundamental findings: pleural sliding; presence of artifacts arising from the pleural line that are generated by the pleura itself (A-lines) or by alterations of the fluid–air composition of the interstitia and alveoli (B-lines); direct visualization of condensed subpleural pulmonary tissue with variable aeration; and detection of pleural effusion.

The first step of LUS is to identify the “bat sign”, corresponding to a hyperechogenic line lying between two adjacent ribs. Such hyperechogenic line corresponds to the pleural line. LUS first evaluation is to check if the pleural line displays lung sliding (the impression of horizontal movement produced by the visceral pleura sliding over parietal pleura during breathing, and represented on M-mode by the “seashore sign”), implying the absence of liquid or air between the two pleural layers at the explored area. Operator should then identify the reverberation artifacts, presenting either as A-lines (horizontal lines parallel to the pleural line and caused by its own reverberation) or B-lines (vertical lines that may be representative of interstitial syndrome) [1, 8, 9].

In adult patients with asthma/chronic obstructive pulmonary disease (COPD) presenting with wheezing, LUS usually shows as an A/nude profile (normal profile, with sliding and A-lines, without any other findings), or at most reveals a decrease in the intensity/absence of pleural sliding due to over-tension. However, this is not only unspecific (as it can be associated with other conditions, most notably pneumothorax), but also very difficult to quantify [1, 10, 11]. Therefore, until this moment, simple point-of-care ultrasonography (POCUS) appeared to be unable to assess the severity of airflow limitation. We present a possible new sonographic method to evaluate airflow impairment and monitoring treatment response.

## Case presentation

We report the case of an obese (BMI of 35) 70-year-old woman, with known history of asthma with frequent exacerbations, in spite of treatment with inhaled corticosteroids and long-acting bronchodilators. She presented in the Emergency Department (ED) breathless, with diffuse wheezing, tachypnea (30/min), room air SpO2 90%, and tachycardia (110 bpm) with normal blood pressure.

**Fig. 1:**
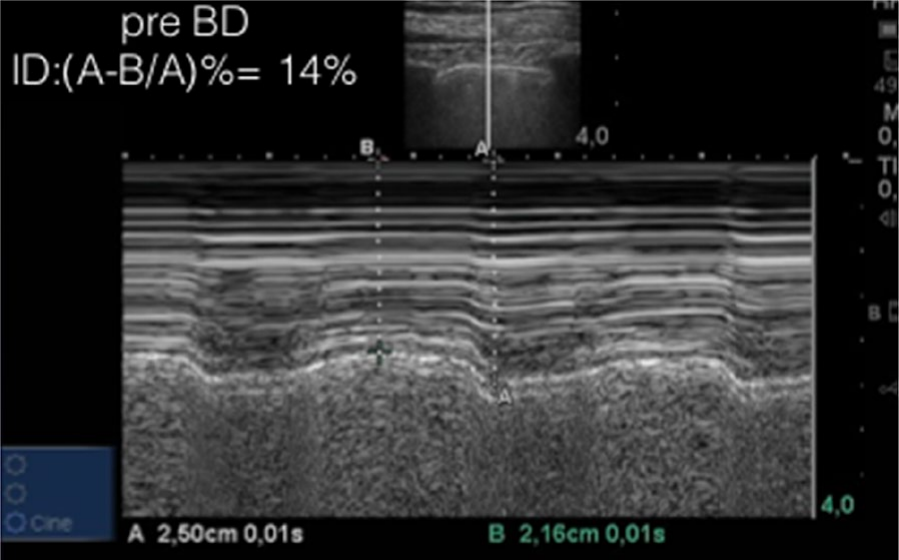
M-mode evaluation of pleural vertical displacement and calculus of the comparison index: $\frac{{{\text{Skin-to-maximal inspiration point distance}}\left( {\text{A}} \right) \, {-}{\text{ skin-to-maximal expiration point distance}}\left( {\text{B}} \right)}}{{{\text{skin-to-maximal inspiration point distance}}\left( {\text{A}} \right)}} \times 100$

A bedside LUS was performed at both apices, with a SONOSITE ^®^ turbo ultrasound system, using a straight linear array probe, with depth setting of 4 cm and soft tissue preset. As the “bat sign” was localized and pleural sliding observed, vertical displacement of the pleural line with each breath (Fig. 1) was noted, probably secondary to hyperinflation and accessory muscle recruitment and its direct effects on parietal pleura. We evaluated the described movement with M-mode and established a comparison index between end-inspiration (A) and end-expiration (B), using the skin as reference:

$$ \frac{{{\text{Skin-to-maximal inspiration point distance}}\left( {\text{A}} \right) \, {-}{\text{ skin-to-maximal expiration point distance}}\left( {\text{B}} \right)}}{{{\text{skin-to-maximal inspiration point distance}}\left( {\text{A}} \right)}} \times 100. $$

**Fig. 2:**
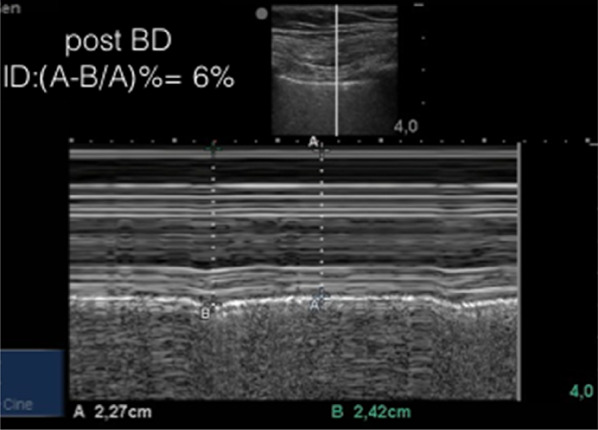
M-mode re-evaluation of the pleural displacement and index calculation at 17 min of treatment, showing improvement with treatment

**Fig. 3:**
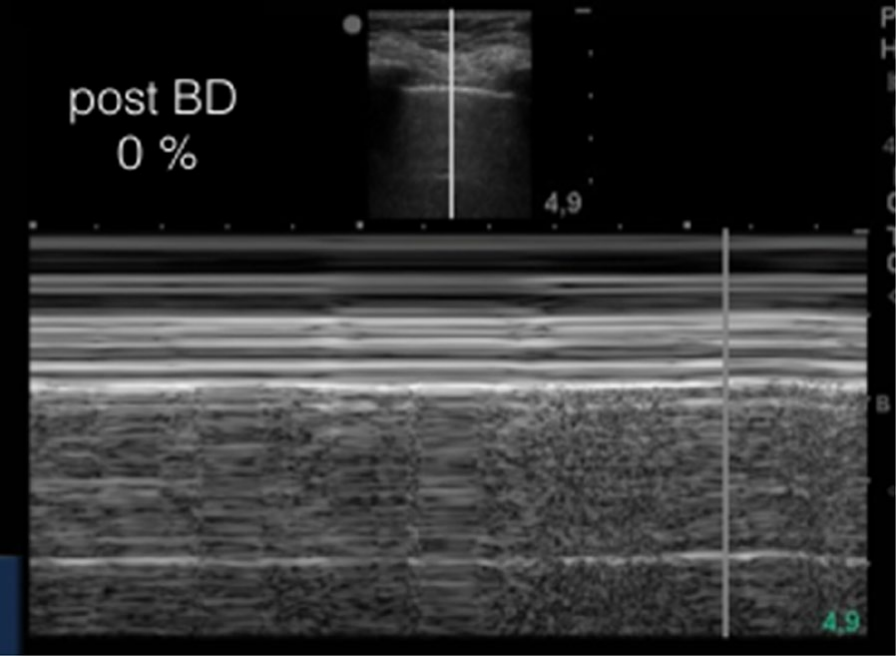
M-mode re-evaluation of the pleural displacement and index calculation at 21 min of treatment, showing complete resolution of pleural displacement an index normalization

The described index measured at admission was 14% (Fig. 1). The patient was then started on usual asthma exacerbation treatment with short-acting bronchodilation and systemic corticosteroids. First re-evaluation, performed at the same point 17 min after treatment administration, showed an index reduction to 6% (Fig. 2). With further treatment, pleural vertical displacement finally disappeared and the index progressed to zero (Fig. 3). Along with the index decrease, symptomatic relief and improved chest auscultation were observed. Peak expiratory flow rate (PEFR) or spirometry were not tested due to lack of patient collaboration, as frequently occurs in the ED.

## Discussion

Asthma and COPD are important causes of morbidity and mortality worldwide. Asthma is characterized by fluctuating symptoms of wheeze, shortness of breath, chest tightness and/or cough and by variable expiratory airflow limitation secondary to airway hyperreactivity and bronchospasm [12]. COPD presents with persistent respiratory symptoms and airflow limitation that is due to airway and/or alveolar abnormalities [13]. Also, asthma/COPD both have in common airflow limitation. During acute exacerbations such flow limitation results in hyperinflation, which seems to be related with sustained post-inspiratory activity of the inspiratory muscles [14]. We hypothesized that hyperinflation and accessory muscle recruitment result in pleural vertical displacement and could explain the findings described. As airflow limitation is reversed, hyperinflation ameliorates and accessory muscles are no longer recruited, thus the pleural vertical displacement will decrease.

We could not find any previous description of such pleural movement in our bibliography research.

Dr. Lichtenstein described two signals detected in M-mode LUS in cases of severe acute dyspnea: the Ifrac and the Nogue-Armendariz phenomena. The Ifrac phenomenon is secondary to accessory respiratory muscle activation, creating a pattern of “muscular sliding” in addition to usual lung sliding. This muscular sliding shows a “seashore pattern” on M-Mode, identifiable above the pleural line, unlike lung sliding that produces a “seashore pattern” under the same line. The Nogue-Armendariz phenomenon represents the rare occurrence of perfect synchrony between such muscular and lung sliding, resulting in a permanent “sand pattern” on M-mode arising at the muscular line and paralleled with the “sand pattern” caused by the movement of the pleural layers [15]. In our case, these phenomena are visible in inspiration during the first evaluation (line A of Fig. 1). Although such findings seem to be present in cases of severe acute dyspnea, they were not correlated with airflow limitation itself and might be difficult to detect in the short time evaluations of the emergency setting.

Some case reports also mentioned absence of B-mode pleural sliding, with loss of its M-mode correspondent “seashore sign” and appearance of “bar-code sign”, in cases of severe airflow impairment [10, 11]. This is also assumed to be a consequence of hyperinflation with pleural over-tension. However, such findings are not specific of those diseases, being more commonly associated with pneumothorax (which can itself present as a complication of severe asthma/COPD exacerbations), but also described in other conditions such as atelectasis, pleural adhesions, severe emphysema or severe fibrosis. Furthermore, a reduction/absence of pleural movement cannot be quantified and therefore would not be suitable to assess the degree of bronchospasm and its response to treatment.

Bronchospasm monitorization is difficult even with standard tests. Although COPD and asthma guidelines underline spirometry and/or peak expiratory respiratory flow (PERF) as pivotal tools for diseases diagnosis and monitorization, they also recognize that those tests show low sensitivity and variation according to age. Also, both techniques need patient collaboration and training to a correct measurement, and PERF monitoring did not prove to ameliorate asthma control in addition to symptom score [12, 16, 17], neither could it predict the need of hospital admissions [18].Those features imply that such complementary tests lack practical applicability in the acute setting; and the American College of Emergency Physicians has already released a statement emphasizing that evidence does not support PERF monitoring for all adult asthma patients [19].

Therefore, currently there is an absence of practical and easily performable tests to diagnose and monitor the airflow limitation, particularly in the emergency setting. The pleural displacement index could be a quick, simple method to indirectly monitor airflow impairment at bedside, independent of patient collaboration.

## Conclusion

New methods for bronchospasm evaluation in the emergency department are needed, and although LUS has been increasingly used as a diagnostic complement there is no description in medical literature of any specific or suggestive sign of severe airflow limitation identifiable with this technique.

We present a pleural vertical displacement index that might represent a new method for monitoring bronchospasm and measuring the severity of asthma/COPD exacerbation. Being a quick, simple and non-invasive test, this could be performed at patient bedside in the ED or during hospitalization. This might allow an easily performable monitorization of airflow limitation and its response to treatment, more practical than PERF, especially in the exacerbated and breathless patient.

LUS evaluation of pleural vertical displacement in the setting of acute airflow impairment will need further validation. How it will present through the severity spectrum of wheezing patients (from not severe exacerbations to imminent respiratory arrest) is a question still to be answered. Also, it is uncertain what is the minimal percentual point difference that translates into a significant clinical improvement or deterioration, and even if these variations occur simultaneously, before or after other clinical signs. Patients with chronic very severe lung hyperinflation may be particularly challenging as pleural vertical displacement may not vary so much during exacerbations. Finally, it is still unclear if it could also be useful in other causes of acute severe dyspnea.

## Data Availability

Data sharing is not applicable to this article as no datasets were generated or analyzed during the current study.

## Abbreviations

COPD: Chronic obstructive pulmonary disease
ED: Emergency department
LUS: Lung ultrasonography
PEFR: Peak expiratory respiratory flow (PERF)
POCUS: Point-of-care ultrasonography
